# Association between mental health comorbidity and health outcomes in type 2 diabetes mellitus patients

**DOI:** 10.1038/s41598-020-76546-9

**Published:** 2020-11-11

**Authors:** Inmaculada Guerrero Fernández de Alba, Antonio Gimeno-Miguel, Beatriz Poblador-Plou, Luis Andrés Gimeno-Feliu, Ignatios Ioakeim-Skoufa, Gemma Rojo-Martínez, Maria João Forjaz, Alexandra Prados-Torres

**Affiliations:** 1 EpiChron Research Group, IIS Aragón, Teaching Unit of Preventive Medicine and Public Health, Zaragoza, Spain; 2 EpiChron Research Group, Aragon Health Sciences Institute (IACS), IIS Aragón, Health Services Research On Chronic Patients Network (REDISSEC), Miguel Servet University Hospital, 50009 Zaragoza, Spain; 3 EpiChron Research Group, IIS Aragón, REDISSEC, University of Zaragoza, Servicio Aragonés de Salud (SALUD), Primary Care Health Centre San Pablo, 50009 Zaragoza, Spain; 4 EpiChron Research Group, IIS Aragón, 50009 Zaragoza, Spain; 5 Regional University Hospital of Málaga, Endocrinology and Nutrition Department, IBIMA, University of Malaga, CIBERDEM, 29010 Málaga, Spain; 6 National Centre of Epidemiology, Institute of Health Carlos III, REDISSEC, 28029 Madrid, Spain

**Keywords:** Diseases, Health care

## Abstract

Type 2 diabetes mellitus (T2D) is often accompanied by chronic diseases, including mental health problems. We aimed at studying mental health comorbidity prevalence in T2D patients and its association with T2D outcomes through a retrospective, observational study of individuals of the EpiChron Cohort (Aragón, Spain) with prevalent T2D in 2011 (n = 63,365). Participants were categorized as having or not mental health comorbidity (i.e., depression, anxiety, schizophrenia, and/or substance use disorder). We performed logistic regression models, controlled for age, sex and comorbidities, to analyse the likelihood of 4-year mortality, 1-year all-cause hospitalization, T2D-hospitalization, and emergency room visit. Mental health comorbidity was observed in 19% of patients. Depression was the most frequent condition, especially in women (20.7% vs. 7.57%). Mortality risk was higher in patients with mental health comorbidity (odds ratio 1.24; 95% confidence interval 1.16–1.31), especially in those with substance use disorder (2.18; 1.84–2.57) and schizophrenia (1.82; 1.50–2.21). Mental health comorbidity also increased the likelihood of all-cause hospitalization (1.16; 1.10–1.23), T2D-hospitalization (1.51; 1.18–1.93) and emergency room visit (1.26; 1.21–1.32). These results suggest that T2D healthcare management should include specific strategies for the early detection and treatment of mental health problems to reduce its impact on health outcomes.

## Introduction

Type 2 diabetes mellitus (T2D) currently represents a significant public health problem worldwide. This chronic multisystem disease results in a progressive deterioration of quality of life^1^, and has been described as one of the most important epidemics of the twenty-first century due to its steadily increasing prevalence^2–4^. According to the International Diabetes Federation, 463 million people worldwide (adults 20–79 years old) were living with T2D in 2019, and this number is expected to increase to 700 million by 2045^4^. T2D is a chronic condition that poses a challenge for patients and their families, caregivers and health systems, due in part to potential complications that may lead to the overutilization of hospital and emergency services.

Rarely appearing in isolation, T2D is frequently accompanied by other chronic diseases; almost 90% of patients with T2D have at least another additional chronic condition (i.e., multimorbidity)^5^. The morbidity burden and the concurrence of certain chronic diseases may increase the risk of adverse health outcomes in T2D patients. The care and healthcare management of this large population group should, therefore, take into account the comorbidity that co-occurs. Conditions such as obesity, high blood pressure and high serum triglycerides are frequently observed in T2D patients as part of the so-called metabolic syndrome^5^. However, diabetes is not only accompanied by metabolic and cardiovascular conditions (i.e., concordant comorbidities of T2D), but also by discordant comorbidities like mental health problems, which originate particularly important adverse effects on the health of T2D patients^1^.

The association between T2D and mental health problems has been well documented^6–13^. The World Health Organization considers that depression is one of the leading causes of health deterioration and progression towards disability^14^; this condition has been associated with a higher risk of diabetes complications and increased health care services utilization among patients with T2D^15,16^. However, most studies published to the date have only focused on specific mental health comorbidities such as depression or anxiety, and less is known about the effect on T2D patients’ health of other kinds of mental health problems like schizophrenia or substance use disorder.

Identifying and treating mental health comorbidities in T2D patients should be a priority^17^. Thus, it is crucial to study how mental health problems affect T2D patients’ health in order to implement more effective diabetes management programmes and improve patients’ health outcomes. This study aimed to explore the prevalence of mental health comorbidty in a Spanish population cohort of T2D patients, and to analyse the specific effect of depression, anxiety, substance use disorder, and schizophrenia on the following T2D outcomes: 4-year all-cause mortality, and 1-year all-cause hospitalization, T2D-hospitalization and emergency room visit.

## Results

The EpiChron Cohort follows 1,070,762 adult users of the public health system of the Spanish region of Aragón. A total of 63,365 adults (46% women, mean age of 69.9 years) in the cohort had a diagnosis of T2D, resulting in a prevalence of 6%. Most of the patients with T2D had at least one more simultaneous chronic disease (Table 1), and approximately one in five individuals (19%) had concurrent mental health comorbidity. The proportion of women was significantly higher in the population with at least one mental health problem than in the group with no mental health comorbidity registered in the health records (62.5% vs. 42.1%, *p* < 0.001). The mean number of chronic comorbidities (excluding mental health ones) was significantly higher in patients with concurrent mental health comorbidities compared with those T2D patients free of mental health problems (4.91 ± 3.02 vs. 3.74 ± 2.55 chronic conditions, *p* < 0.001). More than 90% of patients with T2D and mental health comorbidity had at least two additional comorbidities, and only 2% of them had no other concurrent chronic disease.

**Table 1:** Demographic and clinical characteristics of the population with type 2 diabetes (T2D) based on the presence or not of mental health comorbidity.

Characteristics	Total population (n = 63,365)	Without mental health comorbidity (n = 51,335)	With mental health comorbidity (n = 12,030)	*p* value*
**Sex (n, %)**				< 0.001
Male	34,215 (54.0)	29,707 (57.9)	4508 (37.5)	
Female	29,150 (46.0)	21,628 (42.1)	7522 (62.5)	
**Age (years)**				
Mean age (SD)	69.9 (12.1)	69.8 (12.2)	70.2 (11.8)	0.018
Age groups (n, %)				< 0.001
18–44	1666 (2.6)	1410 (2.75)	256 (2.13)	
45–64	18,445 (29.1)	14,926 (29.1)	3519 (29.3)	
65–74	17,511 (27.6)	14,283 (27.8)	3228 (26.8)	
75–84	19,537 (30.8)	15,654 (30.5)	3883 (32.3)	
≥ 85	6206 (9.8)	5062 (9.9)	1144 (9.6)	
**Additional comorbidities**				
Mean number (SD)	3.96 (2.7)	3.74 (2.6)	4.91 (3.0)	< 0.001
Number (n, %)				< 0.001
0	3020 (4.8)	2746 (5.4)	274 (2.3)	
1	7117 (11.2)	6317 (12.3)	800 (6.7)	
2	10,403 (16.4)	8958 (17.4)	1445 (12.0)	
3	11,184 (17.6)	9405 (18.3)	1779 (14.8)	
4	9668 (15.3)	7818 (15.2)	1850 (15.4)	
5	7487 (11.8)	5835 (11.4)	1652 (13.7)	
≥ 6	14,486 (22.9)	10,256 (20.0)	4230 (35.2)	

*SD* standard deviation.

*p values correspond to the comparison of T2D patients with at least one diagnosis of mental health comorbidity vs. T2D patients with no mental health comorbidity; Chi-squared test and Mann–Whitney U test (non-parametric test) were used.

The most common mental health comorbidities among T2D patients were depression (13.6%) and anxiety (3.17%), both of them more frequent in women (Table 2). Substance use disorder was more frequent in men, mainly in adults up to 64 years old. The prevalence of depression increased with age, while anxiety, substance use disorder and schizophrenia were more frequent in the younger population.

**Table 2:** Frequency and prevalence (%) of mental health comorbidity in the population with type 2 diabetes (n = 63,365) according to sex and age.

Type of mental health comorbidity	Depression	Anxiety	Substance use disorder	Schizophrenia	Total
**Total (n, %)**	8628 (13.6)	2008 (3.2)	1279 (2.0)	931 (1.5)	12,030 (19.0)
**Sex (n, %)**					
Male	2590 (7.6)	730 (2.1)	1125 (3.29)	427 (1.25)	4508 (13.2)
Female	6038 (20.7)	1278 (4.4)	154 (0.53)	504 (1.73)	7522 (25.8)
**Age interval, years (n, %)**					
18–44	129 (7.7)	64 (3.8)	49 (2.9)	51 (3.1)	256 (15.4)
45–64	2178 (11.8)	665 (3.6)	659 (3.6)	365 (2.0)	3519 (19.1)
65–74	2326 (13.3)	533 (3.0)	331 (1.9)	233 (1.3)	3228 (18.4)
75–84	3066 (15.7)	583 (3.0)	206 (1.1)	222 (1.1)	3883 (19.9)
≥ 85	929 (15.0)	163 (2.6)	34 (0.6)	60 (1.0)	1144 (18.4)

The presence of mental health comorbidity was associated with an increased risk of all the T2D outcomes considered in this study. The risk of 4-year all-cause mortality was 1.24 times higher (odds ratio, OR 1.24; 95% confidence interval, CI 1.16–1.31) in patients with at least one concurrent mental health comorbidity, after controlling for sex, age and number of non-mental comorbidities and the presence of the other types of mental health comorbidities (Table 3). The magnitude of this effect was different for each mental health problem. Thus, mortality risk was 2.18 (CI 1.84–2.57) times higher in patients with a diagnosis of substance use disorder, 1.82 (CI 1.50–2.21) times higher in patients with schizophrenia, and 1.14 (CI 1.07–1.22) times higher in those with depression. On the contrary, the likelihood of mortality was not influenced by the presence of anxiety (OR 0.98; CI 0.85–1.13).

**Table 3:** Effect of the presence of mental health comorbidity on 4-year all-cause mortality risk in patients with type 2 diabetes, calculated using two regression analysis models: presence of any mental health comorbidity (Model 1) or type of mental health comorbidity (Model 2).

	Crude OR (95% CI)	Adjusted OR* (95% CI)	*p* value
**Model 1**			
Mental health comorbidity, yes	1.25 (1.19–1.32)	1.24 (1.16–1.31)	< 0.001
Non-mental health comorbidities (number)		1.14 (1.13–1.15)	< 0.001
Sex (Reference: male)		0.58 (0.55–0.60)	< 0.001
Age		1.12 (1.12–1.13)	< 0.001
**Model 2**			
Depression	1.30 (1.23–1.38)	1.14 (1.07–1.22)	< 0.001
Anxiety	0.95 (0.83–1.07)	0.98 (0.85–1.13)	0.769
Substance use disorder	1.30 (1.13–1.50)	2.18 (1.84–2.57)	< 0.001
Schizophrenia	1.17 (0.98–1.39)	1.82 (1.50–2.21)	< 0.001
Non-mental health comorbidities (number)		1.14 (1.13–1.15)	< 0.001
Sex (Reference: male)		0.59 (0.56–0.62)	< 0.001
Age		1.12 (1.12–1.13)	< 0.001

*OR* odds ratio, *CI* confidence interval.

*Adjusted for sex, age, number of non-mental comorbidities, and the presence of the other types of mental health comorbidities.

The simultaneous presence of mental health comorbidity in patients with T2D was associated with a 1.16 (CI 1.10–1.23) times higher risk of 1-year all-cause hospitalization (Table 4). The magnitude of this effect was again different depending on the specific type of mental health comorbidity. The likelihood of all-cause hospitalization was 1.12 (CI 1.05–1.19), 1.40 (CI 1.18–1.66) and 1.58 (CI 1.38–1.81) times higher in patients with depression, schizophrenia and substance use disorder, respectively, whereas it was not associated with the presence of anxiety (OR 1.04; CI 0.92–1.18). We observed similar results for the risk of hospitalization related to T2D, which increased on average 1.51 (CI 1.18–1.93) times when mental health comorbidity was present. Patients with a diagnosis of substance use disorder had the highest risk of T2D-related hospitalization, which was 1.79 (CI 1.05–3.06) times higher, followed by those with depression (OR 1.49; CI 1.14–1.96); whereas anxiety and schizophrenia were not associated with higher risk of T2D-hospitalization. The likelihood of visiting the emergency room was 1.26 (CI 1.21–1.32) times higher when mental health comorbidity was present. The size of this effect was significant for all the specific mental health problems studied, which increased this risk by 22% (OR 1.22; CI 1.16–1.29), 28% (OR 1.28; CI 1.17–1.42), 43% (OR 1.43; CI 1.27–1.61) and 28% (OR 1.28; CI 1.11–1.47) for depression, anxiety, substance use disorder, and schizophrenia, respectively.

**Table 4:** Effect of the presence of mental health comorbidity in patients with type 2 diabetes on 1-year risk of all-cause hospitalization, of T2D-hospitalization and of emergency visit room, calculated using two regression analysis models: presence of any mental health comorbidity (Model 1) or type of mental health comorbidity (Model 2).

	Crude OR (95% CI)	Adjusted OR* (95% CI)	*p* value
**All-cause hospitalization**			
**Model 1**			
Mental health comorbidity, yes	1.35 (1.28–1.42)	1.16 (1.10–1.23)	< 0.001
Non-mental health comorbidities (number)	1.21 (1.20–1.22)	1.19 (1.18–1.20)	< 0.001
Sex (Reference: male)	0.92 (0.88–0.96)	0.71 (0.68–0.74)	< 0.001
Age	1.03 (1.03–1.03)	1.02 (1.02–1.02)	< 0.001
**Model 2**			
Depression	1.33 (1.26–1.41)	1.12 (1.05–1.19)	0.001
Anxiety	1.20 (1.07–1.35)	1.04 (0.92–1.18)	0.518
Substance use disorder	1.79 (1.57–2.04)	1.58 (1.38–1.81)	< 0.001
Schizophrenia	1.26 (1.07–1.49)	1.40 (1.18–1.66)	< 0.001
Non-mental health comorbidities (number)	1.21 (1.20–1.22)	1.19 (1.18–1.20)	< 0.001
Sex (Reference: male)	0.92 (0.88–0.96)	0.72 (0.69–0.76)	< 0.001
Age	1.03 (1.03–1.03)	1.02 (1.02–1.02)	< 0.001
**T2D-hospitalization**			
**Model 1**			
Mental health comorbidity, yes	1.76 (1.39–2.23)	1.51 (1.18–1.93)	0.001
Non-mental health comorbidities (number)	1.18 (1.15–1.21)	1.16 (1.13–1.20)	< 0.001
Sex (Reference: male)	0.90 (0.73–1.12)	0.72 (0.58–0.91)	0.005
Age	1.02 (1.01–1.03)	1.01 (1.00–1.02)	0.111
**Model 2**			
Depression	1.76 (1.35–2.28)	1.49 (1.14–1.96)	0.004
Anxiety	1.55 (0.93–2.56)	1.27 (0.76–2.12)	0.358
Substance use disorder	2.31 (1.37–3.88)	1.79 (1.05–3.06)	0.033
Schizophrenia	1.23 (0.55–2.77)	1.25 (0.55–2.82)	0.592
Non-mental health comorbidities (number)	1.18 (1.15–1.21)	1.16 (1.13–1.20)	< 0.001
Sex (Reference: male)	0.90 (0.73–1.12)	0.73 (0.58–0.92)	0.008
Age	1.02 (1.01–1.03)	1.01 (1.00–1.02)	0.089
**Emergency visit room**			
**Model 1**			
Mental health comorbidity, yes	1.49 (1.43–1.55)	1.26 (1.21–1.32)	< 0.001
Non-mental health comorbidities (number)	1.18 (1.18–1.19)	1.16 (1.16–1.17)	< 0.001
Sex (Reference: male)	1.18 (1.14–1.22)	0.97 (0.93–1.01)	0.100
Age	1.02 (1.02–1.02)	1.01 (1.01–1.01)	< 0.001
**Model 2**			
Depression	1.49 (1.42–1.56)	1.22 (1.16–1.29)	< 0.001
Anxiety	1.49 (1.36–1.64)	1.28 (1.17–1.42)	< 0.001
Substance use disorder	1.54 (1.37–1.72)	1.43 (1.27–1.61)	< 0.001
Schizophrenia	1.23 (1.07–1.42)	1.28 (1.11–1.47)	0.001
Non-mental health comorbidities (number)	1.18 (1.18–1.19)	1.16 (1.16–1.17)	< 0.001
Sex (Reference: male)	1.18 (1.14–1.22)	0.98 (0.94–1.01)	0.193
Age	1.02 (1.02–1.02)	1.01 (1.01–1.01)	< 0.001

*OR* odds ratio, *CI* confidence interval.

*Adjusted for sex, age, number of non-mental comorbidities, and the presence of the other types of mental health comorbidities.

## Discussion

This study shows that approximately one in every five T2D patients has at least one mental health problem (i.e., depression, anxiety, schizophrenia or substance use disorder). Our findings suggest that the presence of mental comorbidity in these patients is associated, to a greater or lesser extent, with an increased risk of adverse health outcomes. Although similar results have been reported in the literature, real-world data in this large-scale population study confirm the significant impact of mental health comorbidity on T2D outcomes.

Several studies have shown that comorbidity is associated with increased mortality in T2D patients and that this increase is higher when psychiatric compared with non-psychiatric comorbidities are present^18,19^. Diabetes represents a significant cause of long-term mortality by itself, and the increased risk of mortality in patients with mental health comorbidity has been well described^19–21^. In our study, a 24% higher likelihood of 4-year mortality observed in patients with mental health comorbidity could be because this kind of comorbidities negatively affects the quality of life and self-care, which can lead to more severe diabetes complications^22^. The negative emotional impact of living with diabetes, known as diabetes distress, has been associated with sub-optimal self-care and glycemic control^23–26^. In addition, some psychiatric drugs such as tricyclic antidepressants can cause metabolic syndrome and exert hyperglycemic effects, exacerbating the progression of T2D^27^.

Various mental health problems have been previously identified as important risk factors associated with poor outcomes in diabetic patients^17,28^. Depression is the most common mental health comorbidity in our study, especially in women, affecting approximately one in ten T2D patients. Depression prevalence has been shown to be higher in patients with T2D than in people free of diabetes; it is greater in women, although the odds ratio for depression in patients with T2D compared with those without is higher in men^29^. It has been discussed that a bidirectional relationship may exist between T2D and depression^28,30–34^. Many studies reported that patients with diabetes have a higher risk of developing depression^22,29,31,33,35^, up to two times higher than in the general population. A recent systematic review underlined that people with depression have a 32% higher risk for developing T2D^36^.

Our study reveals that T2D patients with depression have higher 4-year mortality risk than T2D patients without depression, as well as increased risk for hospitalization related or not to diabetes, and a higher likelihood of using emergency services. Concurrent depression in patients with T2D is associated with poor adherence to treatment, higher complication rates, and increased use of healthcare services^15–17,37^. A significant increase in coronary heart disease and cardiovascular mortality in patients with depression and T2D has also been reported, with significant differences between men and women, suggesting the importance of implementing cardiovascular preventive strategies in this population^38–40^.

Although many patients with diabetes and depression also have anxiety, anxiety can occur in type 1 or type 2 diabetic patients without comorbid depression, especially when diabetes is first diagnosed or when complications first occur^17,41^. In our study, anxiety is more prevalent in women, and its prevalence decreases with age. Anxiety symptoms have been associated with an increased risk of developing incident diabetes^28^; this could be partially due to biological changes (e.g., inflammation, metabolic disorders)^42^, and complex relationships between anxiety and other comorbidities (e.g., depression, obesity). Also, the relationship between diabetes and anxiety is probably bidirectional^43^; however, results are controversial^44,45^. In any case, anxiety is an important comorbidity to consider in people with T2D, as the simultaneous presence of these two conditions is associated with poor glycemic control^46^, obesity^47^, and increased diabetes complications^28,48^. In our study, we found that T2D patients who had anxiety also had a significantly higher risk of visiting an emergency service; however, we did not find significantly increased risk of mortality or hospitalization.

Although less prevalent than depression and anxiety, substance use disorder is the mental health comorbidity in our study with the highest associated risk of mortality, which was increased by 118%, and also of hospitalization (either all-cause or T2D-related) and use of emergency services. Substance use disorder is a disease that leads to an inability to control the use of a legal or illegal drug or medication. It is well known that intravenous drug use is associated with a severe and general deterioration of health outcomes and with an increased likelihood of premature death^49^. However, the specific impact of this mental health problem on T2D patients has not been sufficiently documented, and further longitudinal studies are needed to understand the diabetes onset and outcomes in relation to substance use disorder^50^. Unlike depression and anxiety, in which a bidirectional relationship between them and T2D has been established, substance use disorder has not been clearly identified as a potential cause or consequence of T2D. In any case, our results suggest that substance use disorder should deserve special attention in diabetic patients as it did increase the risk of all-cause hospitalization by 58%, and the risk of T2D-related hospitalization by 79%. This disorder could be especially important in a disease like diabetes, in which appropriate self-care and healthy lifestyles are crucial to avoid complications.

Schizophrenia, the less prevalent mental health comorbidity in our study, is somehow related to diabetes, since T2D has been found to be more prevalent among patients with schizophrenia than in the general population^51^. Some studies consider that schizophrenia itself should be further proposed as a causal factor for T2D due to the strongly demonstrated genetic predisposition to diabetes among people with this mental health problem^52,53^. Our results reveal that this disorder is associated with a higher risk of mortality and all-cause hospitalization. However, its presence was not specifically associated with a greater risk of hospitalization related to T2D. It is well known that antipsychotics are associated with an increased risk of obesity, metabolic syndrome and diabetes mellitus^53^. Excess mortality and all-cause hospitalizations could be explained by aggravating factors for T2D onset and poor diabetes management present in individuals with schizophrenia, such as excessive sedentary lifestyle, social determinants, adverse effects of antipsychotic drugs or limited access to medical care^53,54^.

Diabetes is considered an ambulatory care sensitive condition where effective community care and case management can help prevent the need for hospital admission^55^. However, a poor control/selfcare of the disease potentially due to the presence of mental health comorbidity may lead to an increased risk of unplanned hospitalisations and even of mortality; which could explain in part the results obtained in our study. The high prevalence of comorbidity, specifically of mental health comorbidities, and its negative impact on health outcomes, underscores the importance of promoting continuity of care and of integrated, person-centred care for T2D patients. Active monitoring for signs and symptoms of mental health comorbidities is essential, as is the identification of social circumstances that may influence care seeking, health outcomes, and the need for health services^56^. Our findings are of particular relevance for older populations in which the comorbidity burden is typically higher, and highlight the importance of identifying and adequately treating psychiatric comorbidities that can result in an increased risk of negative health outcomes in T2D patients.

### Strengths and limitations

The main strength of our study is that it is based on a population cohort, including almost all patients with T2D of the reference population in the study area. Data of this cohort are obtained from primary sources of information such as primary care and hospital electronic health records and clinical-administrative databases. This provides a high degree of reliability regarding the diagnosis of T2D and mental health comorbidity; therefore, this information should be more accurate than if it had been self-reported by patients. Our analysis included not only highly prevalent mental health problems such as depression and anxiety, but also other psychiatric disorders less frequently studied in diabetic patients such as schizophrenia and substance use disorder.

On the other hand, one limitation inherent to the analysis of healthcare records is the potential underdiagnosis of certain conditions. We had information on all-cause mortality, but the cause of death was not available in the cohort (e.g., percentage of deaths due to suicide). We neither had the date of diagnosis of T2D or mental health comorbidity, which could bias our results regarding mortality risk by not taking into account the duration of T2D. Furthermore, the risk of mortality may have been overestimated in patients who had T2D for more prolonged periods given the higher likelihood of T2D-associated complications unrelated to the presence of mental health comorbidity.

## Conclusion

Our results indicate that one in five patients with T2D suffers from mental health comorbidity and that the presence of this type of comorbidity is associated with an increased risk of mortality and hospital services use, regardless age, sex and number of other comorbidities. Particular attention should be paid to diabetic patients with substance use disorder or schizophrenia. These findings underline the need for developing global management strategies to facilitate the prevention, early detection, diagnosis and monitoring of mental health comorbidities in T2D patients. The high prevalence of multimorbidity found in T2D patients highlights the importance of providing continuity of care and person-centred approaches to improve the management and outcome of this chronic disease.

## Methods

### Study design, population and data source

This retrospective, observational study was conducted in the EpiChron Cohort^57^. This cohort includes socio-demographic, clinical, health services use and health outcomes information for all users of the public health system of the Spanish region of Aragón (1.3 million inhabitants; 98% of them are users of the public health system). The information contained in electronic health records and clinical-administrative databases is linked at the patient level and then anonymized. A description of the cohort profile, the type of data collected and data curation procedures used has been published elsewhere^57^.

In cohort patients, diagnoses from primary care were coded using the International Classification of Primary Care, First Version (ICPC-1), and those from hospital care were coded using the International Classification of Diseases, Ninth Revision, Clinical Modification (ICD-9-CM). Diagnoses were subsequently grouped in the Expanded Diagnostic Clusters (EDCs) of the Johns Hopkins ACG System (version 11.0, The Johns Hopkins University, Baltimore, MD, US)^58^. This classification system groups clinically similar diagnostic codes, and it is useful in multimorbidity studies to count diseases when, as in this case, diagnoses from different sources and codification systems are used.

**Figure 1:**
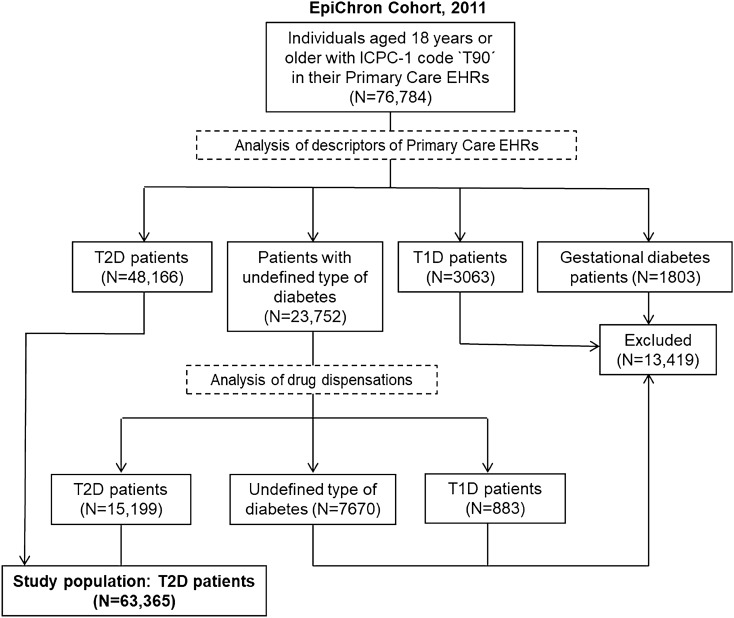
Flow chart of the study population. ICPC-1, International Classification of Primary Care, first version; EHRs, electronic health records; T1D, type 1 diabetes; T2D, type 2 diabetes.

In this study, we used data corresponding to patients of the cohort aged 18 years and older who had either a diagnosis of T2D and/or a pharmaceutical dispensation for T2D treatment in 2011 (Fig. 1). To do this, we selected individuals with an ICPC-1 code ‘T90’ (Diabetes non-insulin dependent) in their primary care health records (n = 76,784). We excluded individuals with an annotation of gestational diabetes (n = 1803) or type 1 diabetes (n = 3063). For patients with unspecified type of diabetes, we selected those with no registered insulin dispensation and having at least one dispensation of sulfonylureas, glucosuric agents, glitazones, and/or dipeptidyl peptidase-4 (DDP-4) inhibitors (n = 15,199). We excluded from the study individuals with a specific treatment for type 1 diabetes (n = 883) and those for whom the type of diabetes could not be determined (n = 7670). Finally, this study included the information of 63,365 TD2 patients.

This study was approved by the Clinical Research Ethics Committee of Aragon (CEICA, PI18/298). The CEICA waived the requirement to obtain informed consent from patients since the information used was anonymised. All research was performed in accordance with the relevant national and international guidelines and regulations, following the Spanish law on the protection of personal data (LOPD 15/1999 of December 14).

### Measurements and outcomes

We classified T2D patients in two groups: patients with no mental health comorbidity, and those with at least one mental health comorbidity defined as the presence of a primary or hospital care diagnosis of depression, anxiety, substance use disorder or schizophrenia, which were identified with EDCs ‘PSY09’, ‘PSY01’, ‘PSY02’, and ‘PSY07’, respectively. The original ICPC-1 and ICD-9 codes conforming each of these EDCs were confirmed and recorded by general practitioners and/or hospital specialists according to specific diagnostic criteria; although part of the diagnoses of mental health comorbidities was confirmed by psychiatrists, we cannot assure that all cases were confirmed or re-diagnosed by a psychiatrist. In Spain, the Diagnostic and Statistical Manual of Mental Disorders, Fifth Edition (DSM-5)^59^ is mostly used by both mental health specialists and general practitioners in clinical practice.

For each patient, we analysed the following explanatory variables: sex, age as of December 31, 2011, number of chronic diseases from the list of 114 EDCs defined by Salisbury et al.^60^, and ‘multimorbidity’, defined as the presence of at least one chronic disease in addition to T2D.

The outcome variables analysed were 4-year all-cause mortality (i.e., from January 1, 2012 to December 31, 2015), and 1-year all-cause hospitalization, T2D-hospitalization, and emergency room visit (i.e., from January 1, 2012 to December 31, 2012). Patients were followed until December 31, 2015, date of death, or date of withdrawal from the cohort (i.e., withdrawal from regional public health system).

### Statistical analysis

We calculated the prevalence of each type of mental health comorbidity in the study population by sex and age group (i.e., 18–44, 45–64, 65–74, 75–84 and ≥ 85 years). We analysed demographic and clinical information of the study population according to the presence or not of mental health comorbidity by means or frequencies/proportions. We compared means using the Mann–Whitney U test, and proportions using the Chi-squared test.

To analyse the effect of the presence of mental health comorbidity on T2D outcomes, we used two logistic regression models and we determined the corresponding odds ratios and 95% confidence intervals, adjusted by sex, age and number of non-psychiatric comorbidities. In the first model, mental health comorbidity was included as a single variable; in the second model, each type of mental health comorbidity was included separately as different variables. Statistical significance was set at *p* < 0.05. We conducted all statistical analyses using Stata (version 11.0, StataCorp LLC, College Station, TX, US).
